# Extreme Weather as an Emerging Public Health Challenge for Youth Sports

**DOI:** 10.5888/pcd23.260476

**Published:** 2026-10-29

**Authors:** Rishin Tandon, Kyle Bunds, Brian P. McCullough

**Affiliations:** 1 Issaquah High School, Issaquah, Washington; 2 Youth Eco Sports Scorecard, Issaquah, Washington; 3 Oklahoma State University, School of Hospitality and Tourism Management, Spears School of Business, Stillwater, Oklahoma; 4 University of Michigan, School of Kinesiology, Sport Management Program, Ann Arbor, Michigan

Every week, more than 60 million young athletes in the US travel to practices, games, and tournaments to play the sports they love (1). Sports support youth development and health in many ways by creating regular opportunities for physical activity, social connection, confidence, discipline, and belonging (1). However, the environmental conditions surrounding youth sports are becoming increasingly difficult to ignore. Extreme heat can make artificial-turf fields dangerous, wildfire smoke can force practices indoors or cancel them entirely, and floods and severe storms can damage the community facilities that local leagues need. As extreme weather events become more frequent, the question is no longer whether they affect youth sports, but how organizations should respond so that athletes can continue to safely play the sports they enjoy and reap the benefits for their health and well-being. These benefits include engaging in regular physical activity, ensuring strong cardiovascular and musculoskeletal health, lowering risk of obesity and other chronic conditions, and improving mental health (2). Thus, youth sports organizations bear a responsibility to incorporate extreme weather considerations into their policies and practices. This responsibility becomes clear through the growing health risks to young athletes, the intensified participation and equity challenges posed by extreme weather events, and the policy and operational decisions organizations must make to maintain safe and accessible youth sport programs in a changing environment.

The NIH Health and Extreme Weather Program Strategic Framework from the National Institutes of Health outlines how exposure to extreme weather affects human health and identifies priorities for research, preparedness, and interventions to reduce these risks (Figure) (3). This framework recognizes that extreme weather can produce both direct and indirect health effects across the lifespan and identifies children and communities with fewer resources as populations at increased risk. Although the framework does not focus on organized sports, youth sports is a setting in which these risks, disruptions, and inequities are visible. Direct effects include heat illness, dehydration, respiratory symptoms, and exposure to unsafe air quality. Indirect effects occur when practices are canceled, fields are damaged, sports seasons become irregular, or children lose access to the physical activity, peer support, and routine that sports typically provide.

**Figure:**
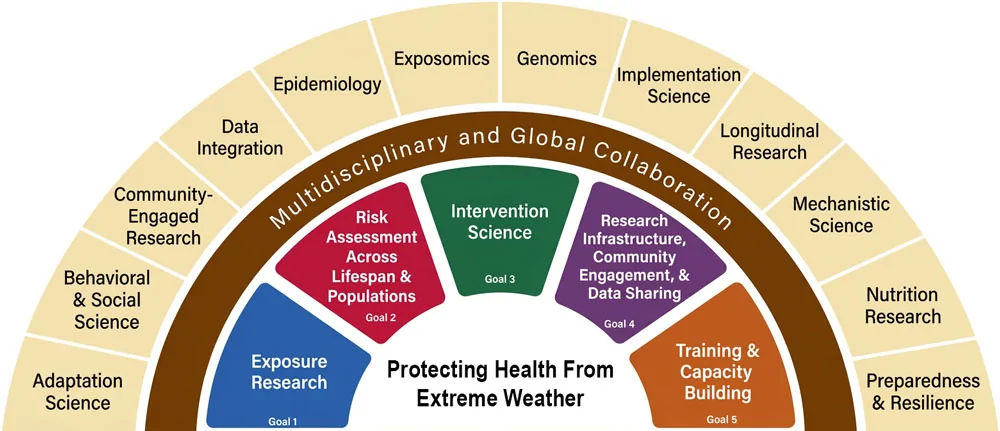
NIH Health and Extreme Weather Program Strategic Framework, National Institutes of Health (3).

The last decade has seen the highest recorded temperatures (4), and according to Climate Central, a Gen Z child experiences 4 times as many extremely hot days as a result of climate change than a Gen X child did (5). Spending time outdoors increases children’s risk of heat-related illness because they breathe more quickly and take in more air in relation to their body weight than adults do (6). With a changing climate, youth athletes lose approximately 1 week of sports participation each year because of climate-related disruptions such as extreme heat and wildfire smoke, which is significant at a population level (7). According to the American Academy of Pediatrics, heat can cause mild heat cramps or heat stroke, which can be fatal (6), and this heat exposure can affect children’s ability to learn and concentrate. These factors move the issue beyond inconvenience, exposing public health concerns well beyond sports. When participation itself becomes a potential health hazard, organizations have a clear duty to intervene proactively by enforcing scheduling changes, heat protocols, and clear standards for safe playing conditions.

Extreme weather events magnify existing structural inequities in youth sports by disproportionately exposing certain groups to environmental risks, thereby limiting equitable access to safe participation. While wealthier travel teams may be able to relocate practices, rent indoor facilities, or travel to unaffected communities after a weather disaster, smaller or less-resourced teams may not have those options. Flooded fields, smoke-filled seasons, or damaged gymnasiums can end participation entirely for children whose families cannot access alternatives. An article in *Time* magazine noted, “practice environments like gyms without air conditioning or turf fields, where temperatures can register 40 degrees hotter than grass, add to the danger” by exposing certain young athletes to potentially detrimental conditions (8). Without intentional intervention, these differences reinforce inequities, turning environmental risks into an issue of disparities based on sociodemographic factors. The repercussions of systemic discriminatory practices such as redlining, the historical practice of denying or limiting housing loans and investment in certain neighborhoods, often communities of color, live on today, as children of color are more likely to live in neighborhoods where they are exposed to more pollution, heat-trapping asphalt, and higher temperatures. Previously redlined areas are also less likely to have access to resources to cope with these extreme weather problems (6). Children living in these neighborhoods are also more likely to face health disparities resulting in disproportionately higher rates of chronic illnesses such as obesity and mental health conditions, which could be at least partially alleviated by more equitable opportunities for physical activity and sports. Youth sports organizations, which often serve as community hubs bringing diverse youth athletes together, are uniquely positioned to help bridge these gaps by providing safer facilities and equitable resource distribution.

Another group that is disproportionately vulnerable to the risks of extreme weather is girls in sports. Research has shown that “adolescent girls experience unique and compounded risks due to physiological differences, sociocultural constraints, and institutional inequities . . . [and] girls’ programs often receive less funding and are deprioritized during climate adaptation efforts” (9). When already underresourced programs are further neglected in extreme weather responses, the gap widens, exacerbating play equity issues. Taken together, these patterns make it clear that extreme weather does not affect all youth athletes equally, and without deliberate action, it will continue to heighten existing health and other inequities.

Because youth sports organizations directly shape training environments, safety standards, and athlete experiences, they have a clear responsibility to proactively protect athlete safety through appropriate policies and coach training. According to the Aspen Institute, only 29% of youth coaches reported receiving training in general injury prevention, and how much of that training covered heat safety specifically is unclear (10). This gap in knowledge reflects a system in which those responsible for athlete safety are not adequately prepared for increasingly harmful environmental conditions. A youth sports coach interviewed in a recent article on extreme heat and young athletes reinforces this through firsthand experience, explaining that he has “never received any training on the risks of heat, early warning signs, or what to do if a player shows symptoms. . . . What we do have are dedicated parents, coaches, and players wanting kids to become the best athletes they can be — and that dedication can be a tool to help ensure safety if we know what to do” (8). These examples reveal that the issue is not a lack of care but inadequate institutional guidance and capacity. Organizations and policymakers could establish required training on heat illness, air-quality risks, hydration, recognition of symptoms, emergency response, and decisions about when conditions are unsafe. Coaches should not have to individually determine whether to cancel or modify activities without clear standards and organizational support.

At the same time, youth sports organizations are positioned to be part of the solution. An analysis of the environmental impact of the sports industry notes that “while the sports industry significantly contributes to global warming, it also has a large potential to be a part of the solution . . . thanks to its broad popularity, sports could be a powerful tool for raising awareness about the climate crisis among people across the world, regardless of their geographical location and social background” (11). The reach of sports is universal, cutting across communities, backgrounds, and age groups, which means youth sports organizations are not only responsible for managing risk, but also capable of influencing behavior at scale and promoting a positive environment and health-related behavior.

While professional, collegiate, and international sport organizations have increasingly recognized the importance of sustainability in sport, including FIFA (Fédération Internationale de Football Association) and the International Olympic Committee, youth sports have not received the same level of attention, even though millions of athletes, parents, coaches, and volunteers participate in this system every week. Initiatives such as the Youth Eco Sports Scorecard (YESS; www.yesscore.org) offer a possible starting point by helping teams focus on practical actions around transportation, waste, energy use, safety, and community engagement (12). Teams can begin by assessing current practices, identifying their most urgent gaps, and selecting realistic actions based on their resources and community needs. YESS encourages youth sports organizations to reduce their environmental footprint while building a culture in which environment and health considerations become part of routine decision-making.

Youth sports have always been about more than just competition; they are environments in which communities gather, where health-promoting habits are formed, and where the next generation learns the power of sports. Therefore, the role of youth sports organizations extends beyond organizing games and practices to protecting athletes and responding to conditions that threaten the health and well-being of youth. Choosing not to prepare exacerbates preventable health risks and inequities. As gyms, rinks, and fields continue to fill with young athletes each weekend, the question becomes harder to ignore: how can youth sports remain an ongoing source of health promotion and community building if we continue to overlook the conditions that are increasingly putting that very experience in jeopardy?

## References

[1] Plassche GC , Miller DJ , Christian RA , Trofa DP , Redler LH , Levine WN , . The preparticipation evaluation of the young athlete — an update to what the orthopedic surgeon needs to know. *Front Sports Act Living.* 2025;7:1650463. 10.3389/fspor.2025.1650463 41195114 PMC12582918

[2] Dhuli K , Naureen Z , Medori MC , Fioretti F , Caruso P , Perrone MA , . Physical activity for health. *J Prev Med Hyg.* 2022;63(2 Suppl 3):E150–E159. 36479484 10.15167/2421-4248/jpmh2022.63.2S3.2756 PMC9710390

[3] National Institutes of Health. NIH Health and Extreme Weather Program Strategic Framework. Accessed June 22, 2026. https://www.niehs.nih.gov/sites/default/files/2025-09/nih-hew-program-strategic-framework_long_508.pdf

[4] Associated Press. Last decade was Earth’s hottest ever as CO2 levels reach an 800,000-year high, says UN report. Accessed September 23, 2026. https://apnews.com/article/climate-change-united-nations-bb8e475cdaa3b3fb348f88276837d50e

[5] Climate Central. Childhood heat risk is rising. Accessed September 23, 2036. https://www.climatecentral.org/climate-matters/childhood-heat-risk-rising-2025

[6] HealthyChildren.org, American Academy of Pediatrics. How climate change, heat and air pollution affect kids’ health. Accessed June 22, 2026. https://www.healthychildren.org/English/safety-prevention/all-around/Pages/how-climate-change-heat-and-air-pollution-affect-kids-health.aspx

[7] Aspen Institute. Project Play. Youth lose one week of sports a year due to climate extremes. Accessed June 22, 2026. https://projectplay.org/news/project-play-survey-youth-lose-one-week-of-sports-a-year-due-to-climate-change

[8] TIME USA. Extreme heat is dangerous for young athletes. Accessed June 22, 2026. https://time.com/7316742/extreme-heat-safety-tips-climate-change-young-athletes/

[9] Hylton-Pelaia J , Dhillon S , Barakat C . Climate change and adolescent girls’ sports: a scoping review and framework-based exploration of emerging barriers and recommendations. *Int J Environ Res Public Health.* 2025;22(12):1764. 10.3390/ijerph22121764 41464398 PMC12732819

[10] Aspen Institute. Project Play. Youth sports facts: benefits. Accessed June 22, 2026. https://projectplay.org/youth-sports/facts/benefits

[11] Dragović B . The influence of the sports industry on climate change and how it can be part of the solution. Earth.Org. December 20, 2022. Accessed June 25, 2026. https://earth.org/sports-climate-change/

[12] Youth Eco Sports Scorecard. Youth Eco Sports Scorecard. Accessed June 22, 2026. https://www.yesscore.org

